# Viral endosymbiotic infection of protozoan parasites: How it influences the development of cutaneous leishmaniasis

**DOI:** 10.1371/journal.ppat.1010910

**Published:** 2022-11-03

**Authors:** Andrea Lafleur, Martin Olivier

**Affiliations:** Department of Microbiology and Immunology, McGill University, and The Research Institute of the McGill University Health Centre, Infectious Diseases and Immunity in Global Health Program, Montréal, Canada; University of Massachusetts, Worcester, UNITED STATES

The pervasiveness of viral infectious agents, capable of parasitizing living organisms all along the evolutionary spectrum, has obligated hosts to evolve defense mechanisms that control viral infection. Elucidation of host–pathogen interactions in model organisms has been fundamental to the translational understanding of human antiviral immunity. Lower organisms such as unicellular prokaryotes and eukaryotes have been shown to act as viral hosts and, in many cases, overcome infection via primitive immune mechanisms. The most widely studied example of this is the CRISPR/Cas system—a restriction mechanism in bacteria and archaea against highly specific viruses known as bacteriophages [1]. This seminal discovery has led to important advances in genetic engineering and has increased interest in viruses that infect unicellular organisms, notably those that are pathogenic to humans, and has led to an emerging role of viruses in this triangular host–pathogen interaction [2].

## Viral infection of lower eukarya: Introduction to endosymbiotic interactions

The first unicellular eukaryotic virus was discovered fortuitously in *Entamoeba histolytica*, after several reports of viral-like particles in electron micrographs of protist ultrastructure [2,3]. Since this discovery, a remarkable variety of viruses has been reported in all major subgroups of unicellular eukaryotic microorganisms [2].

Studies in lower organisms have been key to our current understanding of viral infectious agents. The discovery of nucleocytoplasmic large double-stranded DNA viruses in the amoeba *Acanthamoeba*, for example, shifted the definition of viral genomes to account for increased complexity and genomic plasticity [4].

To enable their survival, pathogens have 2 effective options for infection: The first is to kill their host and spread rapidly, while the second is to coexist within their host. This introduces the novel concept that not all viruses are deleterious to their hosts [5,6]. In fact, many lower eukaryotic viruses are recognized as endosymbionts, often either existing neutrally within their host organisms or providing them with greater resistance to environmental stressors. The mutualistic host–pathogen interaction between the eukaryotic cell and its virus can therefore provide an evolutionary advantage to both organisms, explaining in part the ubiquity of such infections [6]. This mutualism can be illustrated by the yeast *Saccharomyces cerevisiae*, which utilizes toxins of viral endosymbiotic origin to eliminate competing yeast colonies, while being protected through competitive inhibition by its preprotoxin [7]. Thus, the yeast gains a fitness advantage provided by the virus, while the virus can utilize translational machinery [6]. Similar instances of virus-enabled gain of function are omnipresent throughout the biosphere, yet, many of these interactions remain uncharacterized.

### Double-stranded RNA viruses of protozoan parasites: Drivers of pathology

The recent advancement of imaging, molecular, and sequencing technologies has enabled researchers to systematically search for, then characterize, endogenous viruses or virus-like particles in a wide array of unicellular protozoa (Table 1) [8]. This group of parasites is of particular interest as it encompasses several neglected tropical diseases, accounting for millions of new infections, deaths, and disability-adjusted life years worldwide [9].

**Table 1:** Viral endosymbionts of protozoa can affect parasitic pathogenesis.

Protozoon	Viral endosymbiont	Virus type	Impact on parasite	Effect on parasitic pathogenesis	Proposed mechanism of action
*Cryptosporidium* spp.	Cryspovirus (Csp1) [10]	Partitiviridae (dsRNA)	Up-regulation of parasitic fecundity [10].	Increase	Unknown
*Trichomonas vaginalis*	TVV [11]	Totiviridae (dsRNA)	Modulation of parasitic metabolism and immunogenicity [12].	Increase	Interaction with host TLR3 proinflammatory signaling [13]
*Leptomonas seymouri*	Lepsey NLV1 [14]	Narnaviridae (ssRNA+)	Unknown	Unknown	Unknown
*Phytomonas* spp.	PserNV1 [15]	Narnaviridae (ssRNA+)	Unknown	Unknown	Unknown
*Giardia* spp.	GLV [16]	Totiviridae (dsRNA)	Unknown	None	Unknown
*Leishmania (Viannia)*	LRV1 [17]	Totiviridae (dsRNA)	Modulation of parasitic metabolism, virulence, and immunogenicity. [18,19]	Increase	Interaction with host TLR3 proinflammatory signaling and modulation of the NLRP3 inflammasome. [17]
*Leishmania (Leishmania)*	LRV2 [20]	Totiviridae (dsRNA)	Unknown	Unknown	Unknown

dsRNA, double-stranded RNA; GLV, *Giardia lamblia virus*; LRV1, *Leishmania RNA virus 1*; LRV2, *Leishmania RNA virus 2*; NLV1, narna-like virus 1; PserNV1, Pser Narna virus 1; TLR3, Toll-like receptor 3; TVV, *Trichomonas vaginalis virus*.

*Cryptosporidium*, for example, the causative agent of the severe parasitic diarrhoeal infection cryptosporidiosis, can be infected by a bi-segmented double-stranded RNA (dsRNA) viral agent coined *Cryspovirus* (CspV1) that has been positively correlated to parasitic fecundity, underscoring a potential role of the virus in the apicomplexan’s fitness [10].

Among viruses, *Totiviridae* seem to be of particular importance as it encompasses most viral endosymbionts identified in pathogenic protozoa. This viral family has evolved significant diversity, with closely related viruses identified in almost all genera of yeasts, fungi, and protozoa studied [5]. This can be explained, in part, by their circumvention of a lytic infectious phase, in favor of long-term symbiotic persistence [21]. Viruses of this family are non-enveloped and contain uncapped dsRNA genomes. Intriguingly, viral endosymbionts of the *Totiviridae* family have been associated to exacerbated pathology in the context of parasitic infection, despite being noninfectious to mammalian hosts [21].

This phenomenon was first observed in *Trichomonas vaginalis*, the protozoon responsible for the sexually transmitted infection trichomoniasis. Infection of the parasite with *Trichomonas vaginalis virus* (TVV) has been shown to induce differential expression of parasitic virulence factors, notably an increase of the major surface immunogenic virulence factor P270, which aids in evasion of the host immune response [11,12]. TVV has also been reported to induce mammalian host Toll-like receptor 3 (TLR3) proinflammatory signaling, causing exacerbation of trichomoniasis lesions—associated to preterm birth and HIV susceptibility [12,13]. Another example of an infected protozoon is *Giardia*, which causes the parasitic diarrhoeal infection giardiasis and its virus, *Giardia lamblia virus* (GLV) [16]. Similarly, this interaction is persistent, yet no correlation to pathogenesis has been observed.

Flagellates of the *Trypanosomatiadae* family seem to be particularly affected by RNA viral infection, with genetically diverse RNA viruses, including several *Totiviridae*, characterized in *Leptomonas seymouri*, *Phytomonas* spp., and *Leishmania* spp., among others [8,22–24]. While little remains known as to the impact of viral infection on other trypanosomatids, the interaction between *Leishmania RNA virus (LRV)* and *Leishmania* is a flagrant and clinically relevant example of effective mutualism and viral-driven parasitic pathogenicity [2].

### *Leishmania* and *Leishmania RNA virus*: Viral infection and hyperpathogenesis

The vector-borne infection leishmaniasis is caused by the intracellular protozoan *Leishmania*. Characterized as a neglected tropical disease, this sandfly-transmitted infection can present clinically in 3 distinct forms. While cutaneous leishmaniasis (CL) presents as self-limiting dermal lesions, parasites can metastasize to the mucosa, marking progression to the destructive mucocutaneous (MCL) pathological form [25]. Along with the life-threatening and systemic visceral leishmaniasis (VL), these account for an overall annual burden of about 2 million clinical infections and 30,000 deaths [26].

The cytoplasmic viral endosymbiont *LRV*, of the *Totiviridae* family, has been identified as a major driver of leishmaniasis severity and has been associated to progression of CL to MCL, drug treatment failure, and disease relapse [27,28]. The virus is also suspected to contribute to treatment resistance in *Leishmania*/HIV coinfection [2]. *Leishmania RNA virus 1* (LRV1) is frequently identified in species of the *Viannia* subgenus, in particular in *Leishmania v*. *guyanensis* strains endemic to the amazon [29]. This subgenus has highly conserved RNA interference pathways, which was initially thought help establish a balance between viral replication and RNAi-mediated silencing, maintaining viremia under a threshold that would be detrimental to the parasite. However, the more recent discovery of the closely related *Leishmania RNA virus 2* (LRV2) within the *Leishmania Leishmania* subgenera, which lacks functional Argonaute and Dicer, has brought this mechanism into question [27,29,30].

In the absence of its viral endosymbiont, *Leishmania* establishes infection and chronicity through immunogenic silence. With tropism towards macrophages, neutrophils, and dendritic cells, the parasite inhibits NLRP3 inflammasome activation within host cells—an important component of the antiparasitic response [25]. *Leishmania* parasites have evolved multiple synergistic mechanisms for immune evasion via inflammasome inhibition. These include the up-regulation of the host protein A20, which is involved in the inhibition of pro-IL-1β maturation and negative regulation of NF-kB [31]. This will then hinder downstream activation of the IL-1 receptor and the MyD88 adaptor protein, both necessary for triggering parasitotoxic oxidative stress [19]. Other mechanisms include GP63-dependent cleavage of inflammasome components and down-regulation of inflammasome gene transcription, such as caspase-1, which all contribute to the modulation of the host’s antiparasitic response [17,19,32]. Thus, *Leishmania* can circumvent the host immune response long enough to establish a high parasitic load, resulting in tissue damage and lesion progression [25]. In fact, leishmaniasis severity is inversely correlated to inflammasome activation [17].

It follows that LRVs themselves can modulate this immune suppression, capable of dampening inflammasome activation by inhibition of caspase-1 and IL-1β cleavage, while inducing proinflammatory cytokines such as TNFα and IL-12 [17]. Intriguingly, LRVs can simultaneously act as strong innate immunogens, with activation of host endosomal TLR3 signaling in response to the viral dsRNA genome. This triggers TRIF-dependent signaling that contributes to inflammasome inhibition and production of type I IFN, leading to host cell autophagy [17]. In addition, IFN-β production inhibits superoxide-related killing of *Leishmania*, thus conferring additional fitness to the parasite [33]. Therefore, it is LRV that causes the hyperinflammatory phenotype and increased parasitic resistance that is a hallmark of MCL [34].

### LRV transmission and extracellular vesicles: Novel insight into host–pathogen interaction and mechanisms of infection

Extracellular transmission of *Totiviridae* is rare, previously only documented in GLV, with other viruses in the family presumed to be solely transmitted vertically during host cellular division [33]. Recently, however, it has been shown that the viral endosymbiont LRV1 can be horizontally transmitted between *Leishmania* parasites of the *Viannia* subgenus via the parasite’s endosomal sorting complex required for transport (ESCRT) or exosomal pathway [18]. Although utilization of different aspects of the host exosomal pathway had previously been reported in mammalian viruses such as HIV, EBV, and HCV, this discovery is the first of its kind within protozoa [18].

Exosomes are nanosized extracellular vesicles, containing biologically active macromolecules (RNA, proteins, lipids), and are constitutively produced by eukaryotic cells, primarily for intercellular communication [35]. *Leishmania* exosomes themselves have been reported to play an important role in parasitic virulence, notably via their enrichment in the major surface metalloprotease GP63, which influences several key secondary messengers, contributing to pathology via aforementioned signaling cascades [19,35].

**Fig 1:**
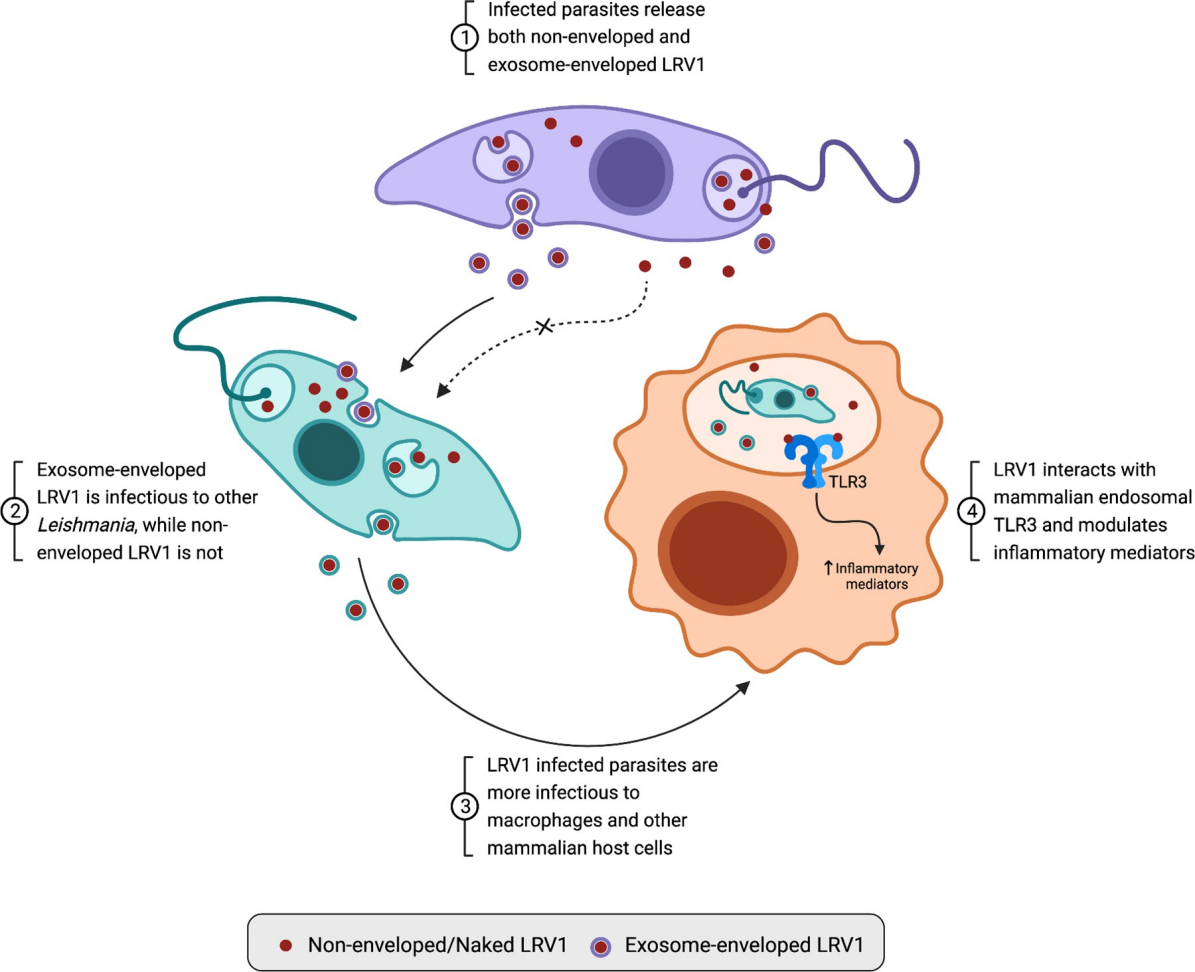
Leishmanial exosomal pathway hijacking by LRV1 enables viral transmission and induces TLR3-mediated hyperpathogenesis in mammalian hosts. LRV1, Leishmania RNA virus 1; TLR3, Toll-like receptor 3.

In the context of LRV1 infection, the virus hijacks the parasite’s exosomal pathway to become encapsulated within an extracellular vesicle, creating a viral envelope that protects the virion from hostile extracellular environments, and enables rapid endocytic uptake by surrounding parasites [25]. Strikingly, viruses shed from the parasite through non-exosomal pathways in the flagellar pocket lack this envelope and are unable to infect other parasites, highlighting the key role of leishmanial exosomes in LRV1 pathogenesis [18] (summarized in Fig 1).

While the natural host of LRV1 is *Leishmania (Viannia) guyanensis*, the exosome-enveloped virus can, in fact, infect and persist within the closely related species *Leishmania (Viannia) panamensis*, while it is rapidly eliminated from the species *Leishmania (Leishmania) mexicana* [18]. The infection of *Leishmania v*. *panamensis* therefore provides a model by which the roles of the virus and the parasite can be untangled in the context of *Leishmania*/LRV1 coinfection. In fact, LRV1-infected *Leishmania panamensis* was shown to induce greater severity of lesions within a murine model, comparatively to its uninfected counterpart, highlighting the role of the virus in leishmanial hyperpathogenesis [25]. Evidence that LRV1 infection modulates the leishmanial translational machinery, effectively hijacking ribosomes, indicates that the virus may play a much more complex role in the parasite’s virulence and fitness [18]. Additionally, this same study showed that LRV1-infected *Leishmania v*. *panamensis* has the capacity to control the virus to undetectable levels over a 10-week time span, suggesting primitive immune machinery [18].

### Future directions in the study of *Leishmania* and *Leishmania RNA virus*

While little is currently known surrounding the evolution of complex antiviral immunity, studying protozoa infected by viral endosymbionts may provide insight ancestral immune mechanisms that enable viral control.

Additionally, increasing treatment resistance for different leishmaniases underscores the need for novel therapies and/or prophylaxis for parasitic infection. Considering the significant burden of LRV-positive *Leishmania* isolates—up to 70% in certain endemic regions—along the virus’ association to treatment failure, LRVs are an obvious target [36]. A significant decrease in leishmaniasis lesion swelling and parasitic load following the immunization of C57BL/6 mice with LRV1 capsid proteins indicates the potential to target a viral endosymbiont to decrease parasitic hyperpathogenicity and generate protective immunity [36]. Thus, this novel vaccine strategy should be further explored as it could potentially be adapted for other *Totiviridae* of pathogenic protozoa.

Overall, investigation of viral endosymbionts of lower eukarya is of utmost clinical and biological importance in the field of infectious disease.
